# Antimicrobial resistance with Streptococcus pneumoniae in the United States, 1997 98.

**DOI:** 10.3201/eid0506.990603

**Published:** 1999

**Authors:** G V Doern, A B Brueggemann, H Huynh, E Wingert

**Affiliations:** Department of Pathology, University of Iowa College of Medicine, Iowa City 52242, USA. gary-doern@uiowa.edu

## Abstract

From November 1997 to April 1998, 1,601 clinical isolates of Streptococcus pneumoniae were obtained from 34 U.S. medical centers. The overall rate of strains showing resistance to penicillin was 29. 5%, with 17.4% having intermediate resistance. Multidrug resistance, defined as lack of susceptibility to penicillin and at least two other non-ss-lactam classes of antimicrobial drugs, was observed in 16.0% of isolates. Resistance to all 10 ss-lactam drugs examined in this study was directly related to the level of penicillin resistance. Penicillin resistance rates were highest in isolates from middle ear fluid and sinus aspirates of children ambulatory-care settings. Twenty-four of the 34 medical centers in this study had participated in a similar study 3 years before. In 19 of these 24 centers, penicillin resistance rates increased 2.9% to 39.2%. Similar increases were observed with rates of resistance to other antimicrobial drugs.


### Antimicrobial Resistance with *Streptococcus pneumoniae* in the United States, 1997–98

**Gary V. Doern, Angela B. Brueggemann, Holly Huynh, Elizabeth Wingert, and Paul Rhomberg**
University of Iowa College of Medicine, Iowa City, Iowa, USA

From November 1997 to April 1998, 1,601 clinical isolates of *Streptococcus pneumoniae* were obtained from 34 U.S. medical centers. The overall rate of strains showing resistance to penicillin was 29.5%, with 17.4% having intermediate resistance. Multidrug resistance, defined as lack of susceptibility to penicillin and at least two other non-ß-lactam classes of antimicrobial drugs, was observed in 16.0% of isolates. Resistance to all 10 ß-lactam drugs examined in this study was directly related to the level of penicillin resistance. Penicillin resistance rates were highest in isolates from middle ear fluid and sinus aspirates of children <5 years of age and from patients in ambulatory-care settings. Twenty-four of the 34 medical centers in this study had participated in a similar study 3 years before. In 19 of these 24 centers, penicillin resistance rates increased 2.9% to 39.2%. Similar increases were observed with rates of resistance to other antimicrobial drugs.

Before 1990, most clinical isolates of *Streptococcus pneumoniae* in the United States were susceptible to a variety of antimicrobial drugs, including penicillin (1,2). In the early 1990s, however, antimicrobial resistance began to emerge (3-7), and ß-lactam resistance as a result of altered penicillin binding proteins was recognized (8-11). Resistance to other non-ß-lactam drugs, such as the macrolides, clindamycin, tetracycline, chloramphenicol, and trimethoprim/sulfamethoxazole (TMP/SMX), began to increase (4-6). Therapeutic failures in patients with pneumococcal infections treated with previously effective drugs were reported (12).

During November 1994 through April 1995, we assessed the prevalence of antimicrobial resistance with *S. pneumoniae* at 30 U.S. medical centers (4). Among 1,527 isolates of *S. pneumoniae*, 14.1% had intermediate resistance, and 9.5% were fully resistant to penicillin. Aggregate rates of intermediate and high penicillin resistance were 2.1% to 52.9%. In addition, high rates of resistance were noted with other antimicrobial drugs.

Address for correspondence: Gary V. Doern, Medical Microbiology Division, C606 GH, Department of Pathology, University of Iowa College of Medicine, Iowa City, IA 52242, USA; fax: 319-356-4916; e-mail: gary-doern@uiowa.edu.

From November 1997 to April 1998, we surveyed 34 U.S. medical centers to assess changes in antimicrobial resistance rates with *S. pneumoniae* during the 3 years since the 1994-95 study. Twenty-four of these centers had also participated in the earlier investigation. Similar patient populations were sampled, and identical test methods were used. In addition, we assessed the relationship between various demographic factors and resistance and undertook a systematic analysis of multidrug resistance. Finally, macrolide and fluoroquinolone resistance was characterized at a molecular level.

### The Study

From November 1, 1997, to April 30, 1998, 1,601 isolates of *S. pneumoniae* were recovered in 34 U.S. medical centers. All isolates included in this study were from consecutive patients. With the exception of specimens from the lower respiratory tract, all isolates were from normally sterile body sites (i.e., blood, cerebrospinal fluid, middle ear fluid, sinus aspirates, pericardial fluid, and pleural fluid). Isolates from lower respiratory tract specimens were included only if they were of clinical significance.

In the study centers, isolates were subcultured onto 5% sheep blood agar plates and incubated overnight at 35°C to 37°C in 5% to 7% $CO_2$. Colony growth was collected on a rayon swab and immediately immersed in a transport tube containing 12 ml of semisolid Ames transport medium with charcoal (Difco Laboratories, Detroit, MI). Transport tubes were then shipped overnight to the University of Iowa College of Medicine for additional analysis (Appendix). The recovery rate from this transport system was 100%. Twelve concentrations each of 23 antimicrobial drugs were tested against 1,601 isolates of *S. pneumoniae*.

Overall, 17.4% of isolates had intermediate and 12.1% had full resistance to penicillin (Table 1). Overall nonsusceptible rates with ceftriaxone and cefuroxime (intermediate plus fully resistant)

Table 1. In vitro activity of 23 antimicrobial agents for 1,601 isolates of *Streptococcus pneumoniae*

| Antimicrobial | Penicillin-susceptible strains (n = 1,127) | | | | | Penicillin-intermediate strains (n = 278) | | | | |
|---|---|---|---|---|---|---|---|---|---|---|
| agent | $MIC_{50}$ | $MIC_{90}$ | MIC range | % I | % R | $MIC_{50}$ | $MIC_{90}$ | MIC range | % I | % R |
| Penicillin | 0.015 | 0.03 | ≤0.004 - 0.06 | -- | -- | 0.5 | 1 | 0.12 - 1 | -- | -- |
| Amoxicillin | 0.015 | 0.03 | ≤0.004 - 0.12 | 0.0 | 0.0 | 0.5 | 2 | 0.015 - 4 | 34.5 | 14 |
| Amox/clav | 0.015 | 0.03 | ≤0.004 - 0.12 | 0.0 | 0.0 | 0.5 | 2 | 0.015 - 4 | 31.7 | 16.9 |
| Ceftriaxone | 0.015 | 0.03 | ≤0.008 - 0.5 | 0.0 | 0.0 | 0.5 | 1 | 0.015 - 4 | 20.1 | 0.7 |
| Cefuroxime | 0.03 | 0.12 | ≤0.015 - 2 | 0.2 | 0.1 | 2 | 4 | 0.12 - 8 | 6.8 | 55.8 |
| Cefpodoxime | 0.03 | 0.06 | ≤0.015 - 4 | – | – | 1 | 4 | 0.03 - 8 | -- | -- |
| Cefprozil | 0.06 | 0.12 | ≤0.03 - 1 | – | – | 2 | 8 | 0.06 - 16 | – | – |
| Cefixime | 0.25 | 0.5 | ≤0.06 - 16 | – | – | 8 | 16 | 0.25 - 32 | – | – |
| Loracarbef | 0.5 | 1 | ≤0.06 - 4 | – | – | 16 | 128 | 0.25 - >128 | – | – |
| Cefaclor | 0.5 | 2 | ≤0.06 - 2 | – | – | 8 | 64 | 0.12 - >128 | – | – |
| Ceftibuten | 4 | 8 | ≤0.25 - >64 | – | – | 64 | >64 | 4 - >64 | – | – |
| Clarithromycin | ≤0.03 | ≤0.03 | ≤0.03 - >64 | 0.5 | 5.2 | ≤0.03 | >64 | ≤0.03 - >64 | 2.2 | 35.3 |
| Erythromycin | 0.06 | 0.06 | ≤0.03 - >64 | 0.3 | 5.7 | 0.06 | >64 | ≤0.03 - >64 | 0.7 | 37.4 |
| Azithromycin | 0.06 | 0.12 | ≤0.03 - >64 | 0.2 | 5.6 | 0.12 | >64 | ≤0.03 - >64 | 0.7 | 37.8 |
| Clindamycin | 0.06 | 0.06 | ≤0.008 - >8 | 0.1 | 1.1 | 0.06 | >8 | ≤0.008 - 8 | 0.0 | 12.9 |
| Trovafloxacin | 0.06 | 0.12 | 0.015 - 8 | 0.0 | 0.2 | 0.06 | 0.12 | 0.015 - 0.25 | 0.0 | 0.0 |
| Tetracycline | 0.12 | 0.25 | ≤0.03 - 64 | 0.2 | 2.5 | 0.25 | 32 | ≤0.03 - >64 | 0.7 | 27.7 |
| TMP/SMX | 0.12 | 1 | 0.06 - 32 | 6.5 | 5.9 | 2 | 8 | ≤0.03 - >32 | 19.8 | 42.4 |
| Chloramphenicol | 2 | 4 | ≤0.5 - 16 | – | 0.8 | 4 | 16 | ≤0.05 - 16 | – | 11.9 |
| Rifampin | ≤0.12 | ≤0.12 | ≤0.12 - 0.5 | 0.0 | 0.0 | ≤0.12 | ≤0.12 | ≤0.12 - 2 | 0.7 | 0.0 |
| Linezolid[a] | 1 | 2 | 0.12 - 2 | – | – | 1 | 2 | 0.25 - 2 | – | – |
| Quin/dalfo | 0.25 | 0.5 | 0.06 - 8 | 0.0 | 0.2 | 0.25 | 0.5 | 0.06 - 1 | 0.0 | 0.0 |
| Vancomycin | 0.25 | 0.5 | 0.03 - 0.5 | 0.0 | 0.0 | 0.25 | 0.5 | 0.06 - 0.5 | 0.0 | 0.0 |

| Antimicrobial | Penicillin-resistant strains (n = 196) | | | | | All strains (n = 1,601) | | | | |
|---|---|---|---|---|---|---|---|---|---|---|
| agent | $MIC_{50}$ | $MIC_{90}$ | MIC range | % I | % R | $MIC_{50}$ | $MIC_{90}$ | MIC range | % I | % R |
| Penicillin | 2 | 4 | 2 - 8 | -- | -- | 0.015 | 2 | ≤0.004 - 8 | 17.4 | 12.1 |
| Amoxicillin | 2 | 8 | 1 - 8 | 9.7 | 90.3 | 0.03 | 2 | ≤0.004 - 8 | 7.2 | 13.5 |
| Amox/clav | 2 | 8 | 1 - 8 | 6.6 | 93.4 | 0.03 | 2 | ≤0.004 - 8 | 6.3 | 14.4 |
| Ceftriaxone | 1 | 2 | 0.5 - 8 | 68.4 | 31.6 | 0.03 | 1 | ≤0.008 - 8 | 10.9 | 4 |
| Cefuroxime | 4 | 8 | 2 - 32 | 0.0 | 100 | 0.03 | 4 | ≤0.015 - 32 | 1.3 | 22 |
| Cefpodoxime | 4 | 8 | 1 - >32 | -- | -- | 0.06 | 4 | ≤0.05 - >32 | – | -- |
| Cefprozil | 8 | 16 | 2 - 64 | – | – | 0.12 | 8 | ≤0.03 - 64 | – | -- |
| Cefixime | 32 | 64 | 2 - 128 | – | – | 0.25 | 16 | ≤0.06 - 128 | – | -- |
| Loracarbef | 128 | >128 | 32 - >128 | – | – | 1 | 128 | ≤0.06 - >128 | – | -- |
| Cefaclor | 128 | >128 | 16 - >128 | – | – | 0.5 | 64 | ≤0.06 - >128 | – | -- |
| Ceftibuten | >64 | >64 | ≤16 - >64 | – | – | 44 | >64 | ≤0.25 - >64 | – | – |
| Clarithromycin | 2 | >64 | ≤0.03 - >64 | 3.6 | 64.8 | ≤0.03 | 4 | ≤0.03 - >64 | 1.2 | 17.7 |
| Erythromycin | 4 | >64 | ≤0.03 - >64 | 0.5 | 68.4 | 0.06 | 8 | ≤0.03 - >64 | 0.4 | 18.9 |
| Azithromycin | 8 | >64 | ≤0.03 - >64 | 1.5 | 67.3 | 0.12 | 16 | ≤0.03 - >64 | 0.4 | 18.7 |
| Clindamycin | 0.06 | >8 | ≤0.008 - >8 | 0.5 | 21.4 | 0.06 | 0.06 | ≤0.008 - >8 | 0.1 | 5.6 |
| Trovafloxacin | 0.06 | 0.12 | 0.03 - 4 | 0.5 | 0.5 | 0.06 | 0.12 | 0.015 - 8 | 0.1 | 0.2 |
| Tetracycline | 16 | 32 | 0.06 - 64 | 0.5 | 51.5 | 0.12 | 16 | ≤0.03 - >64 | 0.3 | 12.9 |
| TMP/SMX | 4 | 16 | 0.06 - 32 | 21.9 | 71.9 | 0.25 | 4 | ≤0.03 - 32 | 10.7 | 20.4 |
| Chloramphenicol | 4 | 16 | ≤0.5 - >16 | – | 37.2 | 2 | 4 | ≤0.5 - >16 | – | 7.2 |
| Rifampin | ≤0.12 | ≤0.12 | ≤0.12 - >4 | 0.0 | 0.5 | ≤0.12 | ≤0.12 | ≤0.12 - >4 | 0.1 | 0.1 |
| Linezolid | 1 | 2 | 0.5 - 2 | – | – | 1 | 2 | 0.12 - 2 | – | -- |
| Quin/dalfo | 0.25 | 0.5 | 0.12 1 | 0.0 | 0.0 | 0.25 | 0.5 | 0.06 - 8 | 0.0 | 0.1 |
| Vancomycin | 0.25 | 0.5 | 0.06 - 0.5 | 0.0 | 0.0 | 0.25 | 0.5 | 0.03 - 0.5 | 0.0 | 0.0 |

[a]Because of the lack of NCCLS breakpoints for linezolid, resistance rates were not determined.
Amox/clav, amoxicillin/clavulanate; TMP/SMX, trimethoprim/sulfamethoxazole; Quin/dalfo, quinupristin/dalfopristin.
MIC, minimum inhibitory concentration; I, intermediate resistance; R, resistant.

were 14.9% and 23.3%, respectively. Because National Committee for Clinical Laboratory Standards (NCCLS)-approved breakpoints are lacking for the six other cephalosporins examined in this study (cefpodoxime, cefixime, ceftibuten, cefprozil, cefaclor, and loracarbef), rates of resistance were not determined for these drugs. However, when MIC values were compared, cefpodoxime was the most active.

Comparison of MIC values of the three macrolides we examined showed that clarithromycin was consistently twice as active as erythromycin, which in turn was consistently twice as active as azithromycin (Table 1). Overall rates of resistance, however, based on NCCLS breakpoints, which differ for these agents, were similar (18%-19%).

Compared with erythromycin as an indicator of macrolide activity, 302 (18.9%) isolates had MICs ≥1 µg/ml and thus were classified as resistant (Table 1). Among these, 217 (71.9%) had erythromycin MICs ≤32 µg/ml; the remaining 85 strains (28.1%) had erythromycin MICs ≥64 µg/ml. Of the 217 strains with erythromycin MICs ≤32, 214 had clindamycin MICs ≤0.25 and thus were categorized as clindamycin susceptible. Thirty-five of these strains, randomly selected, were examined by polymerase chain reaction (PCR) for the presence of *ermAM* and *mefE* genes. Of the 85 strains with erythromycin MICs ≥64 µg/ml, 83 had clindamycin MICs ≥8. Thirty-eight of these isolates, chosen randomly, were *ermAM* positive; 12 were also positive for *mefE* (Table 2). Five resistant strains were also characterized for the presence of macrolide resistance determinants (Table 2). Finally, the three isolates (Table 2) negative for both the *ermAM* and *mefE* genes were also negative by PCR for other known determinants of macrolide/lincosamide resistance in gram-positive bacteria (*ermA, ermC, ereA, ereB, msrA*, and *linA* genes).

Of the 1,601 isolates examined, one had intermediate resistance to trovafloxacin with an MIC 2 µg/ml; three strains (0.2%) were resistant, two with trovafloxacin MICs 4 µg/ml and one with a trovafloxacin MIC 8 µg/ml. The single strain with intermediate resistance had a ciprofloxacin MIC of 16 and an asp83→tyr substitution in the C subunit of topoisomerase IV, as well as a ser84→tyr substitution in the A subunit of DNA gyrase. The three trovafloxacin-resistant strains had ciprofloxacin MICs ≥32 µg/ml and a ser79→phe substitution in the C subunit of topoisomerase, as well as a ser84→phe substitution in the A subunit of DNA gyrase. None of these four strains had detectable mutations in the QRDR of *par E*.

Overall rates of resistance, expressed as the percentage of isolates intermediate or resistant, for selected other agents are described in Table 1: tetracycline, 13.2%, TMP/SMX, 31.1%, chloramphenicol, 7.2%, and rifampin, 0.2%. Linezolid was uniformly active over a narrow range of MICs (i.e., 0.12 to 2 µg/ml). Two strains among the 1,601 examined in this study were resistant to quinupristin/dalfopristin; one had an MIC 4 µg/ml and the other 8 µg/ml. Vancomycin was uniformly active against the 1,601 isolates of *S. pneumoniae* in this survey, with MICs ≤0.5 µg/ml.

The in vitro activity of all ß-lactams (penicillins, ß-lactamase inhibitor combinations and cephalosporins), macrolides, clindamycin, tetracycline, TMP/SMX, and chloramphenicol was lowest with high-level penicillin-resistant strains of *S. pneumoniae* and greatest with penicillin-susceptible isolates. This trend was not apparent with linezolid, trovafloxacin, rifampin, quinupristin/dalfopristin, or vancomycin.

The prevalence of resistance to selected agents was assessed according to the specimen

Table 2. Characterization of 302 *Streptococcus pneumoniae* isolates that were erythromycin resistant (MICs 1≥ µg/ml)

| Erythromycin MIC | No. of strains | No. with clindamycin MICs of 0.25 | 0.5 | 1 | 2 | 8 |
|---|---|---|---|---|---|---|
| 1 | 15 | 15[a] | 0 | 0 | 0 | 0 |
| 2 | 52 | 51[b] | 0 | 0 | 0 | 1[c] |
| 4 | 53 | 53[d] | 0 | 0 | 0 | 0 |
| 8 | 68 | 68[e] | 0 | 0 | 0 | 0 |
| 16 | 23 | 22[f] | 1[g] | 0 | 0 | 0 |
| 32 | 6 | 5[h] | 0 | 0 | 0 | 1[i] |
| 64 | 85 | 0 | 0 | 1[j] | 1[k] | 83[l] |

[a]Four isolates characterized for *ermAM* and *mefE*; all four *ermAM-/mefE+*.
[b]Seven isolates characterized; all seven *ermAM-/mefE+*.
[c]This isolate was *ermAM+/mefE-*.
[d]Seven isolates characterized; one *ermAM-/mefE-*; six *ermAM-/mefE+*.
[e]Seven isolates characterized; all seven *ermAM-/mefE+*.
[f]Four isolates characterized; all four *ermAM-/mefE+*.
[g]This isolate was *ermAM-/mefE-*.
[h]Three isolates characterized; all *ermAM-/mefE+*.
[i]This isolate was *ermAM+/mefE-*.
[j]This isolate was *ermAM-/mefE+*.
[k]This isolate was *ermAM-/mefE-*.
[l]Thirty-eight of these isolates were characterized; 26 were *ermAM+/mefE-*; 12 were *ermAM+/mefE+*.

source of isolates, the patient's age, and the health-care setting (Table 3). In general, the highest resistance rates for all antimicrobial drugs were observed in middle ear fluid and sinus aspirate isolates and in isolates from patients ≤5 years old and from patients seen in ambulatory-care settings.

When patterns of multidrug resistance were analyzed, coresistance to penicillin, the macrolides, tetracycline, TMP/SMX, and chloramphenicol was observed in 5.4% of isolates (Table 4). Resistance to the first four of these drugs but not to chloramphenicol was seen with 3.7% of strains. The most common pattern of multidrug resistance (intermediate level or resistant to penicillin and resistant to TMP/SMX, but susceptible to the macrolides, tetracyclines, and chloramphenicol) was observed in 6.9% of isolates.

In the 34 participating medical centers, the lowest and highest overall rates of penicillin resistance (intermediate plus resistant) were 12.8% and 64.6% (Table 5). Eight medical centers had 10% to 18% penicillin resistance; 11 centers had 19% to 26%; 5 had 27% to 36%; 7 had 37% to 45%; 3 had >46%. Although no distinct geographic clustering was identified, the highest overall rates of penicillin resistance were in the

Table 4. Frequency of isolation of strains of *Streptococcus pneumoniae* with various patterns of antimicrobial resistance[a]

| Pattern of resistance[b] | | | | | |
|---|---|---|---|---|---|
| Penicillin | Erythromycin | Tetracycline | TMP/SMX | Chloramphenicol | No. |
| R | S | S | S | S | 94 |
| R | S | S | R | S | 111 |
| R | S | S | R | R | 1 |
| R | S | R | S | S | 4 |
| R | S | R | R | S | 8 |
| R | S | R | R | R | 15 |
| R | R | S | S | S | 9 |
| R | R | S | R | S | 75 |
| R | R | S | R | R | 3 |
| R | R | R | S | S | 10 |
| R | R | R | R | S | 57 |
| R | R | R | R | R | 87 |

[a]Total number of isolates tested = 1,601.
[b]Resistance includes both intermediate and resistant strains.
R, resistant; S, susceptible; TMP/SMX, trimethoprim/sulfamethoxazole.

Table 3. Recovery of *Streptococcus pneumoniae* strains with intermediate and high levels of resistance, by specimen source and patient characteristics

| | | No. (%) of resistant isolates for the following antimicrobial drugs | | | | | | | | | | | |
|---|---|---|---|---|---|---|---|---|---|---|---|---|---|
| | Total no. | Penicillin | | Ceftriaxone | | Erythromycin | | Tetracycline | | TMP/SMX | | Chloramphenicol | |
| Characteristic | of isolates | I | R | I | R | I | R | I | R | I | R | I | R |
| Specimen source[a] | | | | | | | | | | | | | |
| LRT | 773 | 144 | 103 | 91 | 36 | 5 | 152 | 1 | 116 | 89 | 149 | -- | 67 |
| | | (18.6) | (13.3) | (11.8) | (4.7) | (0.6) | (20.9) | (0.1) | (15.0) | (11.5) | (19.3) | | (8.7) |
| Blood | 509 | 68 | 41 | 35 | 16 | 1 | 59 | 2 | 33 | 40 | 87 | -- | 17 |
| | | (13.4) | (8.1) | (6.9) | (3.1) | (0.2) | (11.6) | (0.4) | (6.5) | (7.9) | (17.1) | | (3.3) |
| URT | 238 | 50 | 48 | 44 | 11 | 0 | 77 | 2 | 53 | 28 | 74 | -- | 28 |
| | | (21.0) | (20.2) | (18.5) | (4.6) | (0.0) | (32.4) | (0.8) | (22.3) | (11.8) | (31.1) | | (11.8) |
| BF/CSF | 60 | 12 | 3 | 4 | 1 | 0 | 9 | 0 | 3 | 1 | 13 | -- | 2 |
| | | (20.0) | (5.0) | (6.7) | (1.7) | (0.0) | (15.0) | (0.0) | (5.0) | (1.7) | (21.7) | | (3.3) |
| Other | 21 | 4 | 1 | 1 | 0 | 0 | 5 | 0 | 1 | 2 | 3) | -- | 1 |
| | | (19.0) | (4.8) | (4.8) | (0.0) | (0.0) | (23.8) | (0.0) | (4.8) | (9.5) | (14.3) | | (4.8) |
| Age group (years) | | | | | | | | | | | | | |
| ≤5 | 432 | 88 | 79 | 72 | 23 | 1 | 115 | 3 | 71 | 62 | 128 | -- | 42 |
| | | (20.4) | (18.3) | (16.7) | (5.3) | (0.2) | (26.6) | (0.7) | (16.4) | (14.4) | (29.6) | | (9.7) |
| 6-20 | 97 | 14 | 6 | 6 | 1 | 0 | 12 | 0 | 11 | 11 | 14 | -- | 4 |
| | | (14.4) | (6.2) | (6.2) | (1.0) | (0.0) | (12.4) | (0.0) | (11.3) | (11.3) | (14.4) | | (4.1) |
| 21-50 | 407 | 76 | 42 | 39 | 18 | 2 | 71 | 0 | 56 | 32 | 80 | -- | 22 |
| | | (18.7) | (10.6) | (9.6) | (4.4) | (0.5) | (17.4) | (0.0) | (13.8) | (7.9) | (19.7) | | (5.4) |
| >50 | 652 | 96 | 68 | 57 | 22 | 3 | 102 | 2 | 66 | 65 | 102 | -- | 46 |
| | | (14.7) | (10.4) | (8.7) | (3.4) | (0.5) | (15.6) | (0.3) | (10.1) | (10.0) | (15.6) | | (7.1) |
| Service | | | | | | | | | | | | | |
| Inpatient | 969 | 172 | 111 | 104 | 37 | 3 | 171 | 3 | 107 | 85 | 192 | -- | 61 |
| | | (17.8) | (11.5) | (10.7) | (3.8) | (0.3) | (17.6) | (0.3) | (11.0) | (8.8) | (19.8) | | (6.3) |
| Outpatient | 628 | 106 | 85 | 71 | 27 | 3 | 131 | 2 | 99 | 86 | 134 | -- | 54 |
| | | (16.9) | (13.5) | (11.3) | (4.3) | (0.3) | (20.9) | (0.3) | (15.8) | (13.7) | (21.3) | | (8.6) |

[a]BF/CSF, body fluids/cerebrospinal fluid; LRT, lower respiratory tract; URT = upper respiratory tract.
TMP/SMX, trimethoprim/sulfamethoxazole.

Table 5. Resistance rates of selected antimicrobial drugs, by study center

| | | Peni- cillin | | Ceftria- xone | | Erythro- mycin | | Tetra- cycline | | Chloram- phenicol | | TMP/ SMX | |
|---|---|---|---|---|---|---|---|---|---|---|---|---|---|
| Medical center and location | No. of isolates | % I | % R | % I | % R | % I | % R | % I | % R | % I | % R | % I | % R |
| Children's Hospital & Medical Center, Seattle, WA | 50 | 26.0 | 12.0 | 10.0 | 8.0 | 0.0 | 30.0 | 0.0 | 24.0 | -- | 10.0 | 8.0 | 34.0 |
| Veteran's Affairs Medical Center, Portland, OR | 24 | 12.5 | 4.2 | 8.3 | 0.0 | 0.0 | 12.5 | 0.0 | 12.5 | -- | 0.0 | 4.2 | 25.0 |
| UCSF Medical Center, San Francisco, CA | 47 | 4.3 | 8.5 | 6.4 | 4.3 | 0.0 | 12.8 | 2.1 | 14.9 | -- | 6.4 | 6.4 | 19.1 |
| UCLA Medical Center, Los Angeles, CA | 55 | 27.3 | 14.5 | 20.0 | 1.8 | 1.8 | 18.2 | 0.0 | 20.0 | -- | 10.9 | 25.5 | 21.8 |
| Pathology Medical Laboratories, San Diego, CA | 51 | 15.7 | 2.0 | 3.9 | 0.0 | 0.0 | 13.7 | 0.0 | 11.8 | -- | 2.0 | 9.8 | 9.8 |
| University of Utah Medical Center, Salt Lake City, UT | 53 | 13.2 | 9.4 | 9.4 | 1.9 | 0.0 | 17.0 | 0.0 | 7.5 | – | 5.7 | 13.2 | 20.8 |
| Denver Health, Denver, CO | 26 | 7.7 | 7.7 | 11.5 | 0.0 | 0.0 | 7.7 | 0.0 | 11.5 | -- | 11.5 | 3.8 | 15.4 |
| Good Samaritan Medical Center, Phoenix, AZ | 64 | 11.1 | 29.6 | 24.1 | 11.1 | 0.0 | 35.2 | 0.0 | 20.4 | -- | 5.6 | 11.1 | 38.9 |
| University Hospital, Albuquerque, NM | 51 | 11.8 | 5.9 | 5.9 | 0.0 | 0.0 | 13.7 | 0.0 | 7.8 | -- | 3.9 | 9.8 | 11.8 |
| Texas Children's Hospital, Houston, TX | 48 | 39.6 | 25.0 | 26.0 | 10.4 | 0.0 | 43.8 | 2.1 | 22.9 | -- | 14.6 | 20.8 | 41.7 |
| Parkland Health & Hospital System, Dallas, TX | 36 | 19.4 | 11.1 | 11.1 | 2.8 | 0.0 | 27.8 | 0.0 | 22.2 | -- | 11.1 | 16.7 | 22.2 |
| Mayo Clinic, Rochester, MN | 48 | 16.7 | 6.3 | 6.3 | 4.2 | 0.0 | 20.8 | 2.1 | 10.4 | -- | 6.3 | 10.4 | 16.7 |
| University of Iowa Hospitals and Clinics, Iowa City, IA | 49 | 16.3 | 22.4 | 16.3 | 4.1 | 0.0 | 22.4 | 0.0 | 20.4 | -- | 12.2 | 14.3 | 22.4 |
| Children's Hospital, Milwaukee, WI | 55 | 12.7 | 7.3 | 7.3 | 3.6 | 0.0 | 10.9 | 1.8 | 5.5 | -- | 3.6 | 12.7 | 14.5 |
| Evanston Hospital, Evanston, IL | 35 | 11.4 | 2.9 | 2.9 | 0.0 | 0.0 | 14.3 | 0.0 | 5.7 | -- | 0.0 | 5.7 | 8.6 |
| Rush-Presbyterian St. Luke's Medical Center, Chicago, IL | 41 | 14.6 | 4.9 | 4.9 | 2.4 | 0.0 | 19.5 | 0.0 | 17.1 | -- | 7.3 | 2.4 | 19.5 |
| Clarian Health Methodist Hospital, Indianapolis, IN | 55 | 18.2 | 7.3 | 3.6 | 0.0 | 1.8 | 16.4 | 0.0 | 7.3 | -- | 3.6 | 10.9 | 12.7 |
| Washington University, St. Louis, MO | 55 | 12.7 | 16.4 | 14.5 | 3.6 | 0.0 | 12.7 | 0.0 | 9.1 | -- | 5.5 | 18.2 | 12.7 |
| Henry Ford Health System, Detroit, MI | 60 | 21.7 | 8.3 | 10.0 | 3.3 | 0.0 | 10.0 | 0.0 | 8.3 | -- | 6.7 | 15.0 | 10.0 |
| The Cleveland Clinic Foundation, Cleveland, OH | 60 | 10.0 | 13.3 | 11.7 | 3.3 | 0.0 | 20.0 | 1.7 | 10.0 | -- | 6.7 | 11.7 | 15.0 |
| Temple University Hospital, Philadelphia, PA | 42 | 7.1 | 14.3 | 4.8 | 9.5 | 2.4 | 9.5 | 0.0 | 7.1 | -- | 4.8 | 2.4 | 9.5 |
| Geisinger Medical Center, Danville, PA | 49 | 30.6 | 8.2 | 12.2 | 0.0 | 0.0 | 20.4 | 0.0 | 20.4 | -- | 12.2 | 10.2 | 14.3 |
| SUNY Health Science Center, Syracuse, NY | 50 | 12.0 | 8.0 | 8.0 | 2.0 | 0.0 | 8.0 | 0.0 | 8.0 | -- | 6.0 | 12.0 | 8.0 |
| University of Rochester Medical Center, Rochester, NY | 50 | 10.0 | 10.0 | 10.0 | 2.0 | 2.0 | 10.0 | 0.0 | 8.0 | -- | 6.0 | 8.0 | 14.0 |
| Columbia Presbyterian Medical Center, New York, NY | 53 | 18.9 | 1.9 | 3.8 | 0.0 | 0.0 | 3.8 | 0.0 | 7.5 | -- | 5.7 | 5.7 | 7.5 |
| Dartmouth Hitchcock Medical Center, Lebanon, NH | 50 | 8.0 | 8.0 | 4.0 | 4.0 | 0.0 | 10.0 | 0.0 | 6.0 | -- | 6.0 | 6.0 | 22.0 |
| Hartford Hospital, Hartford, CT | 51 | 17.6 | 9.8 | 9.8 | 0.0 | 2.0 | 5.9 | 0.0 | 7.8 | -- | 2.0 | 11.8 | 17.6 |
| Beth Israel Deaconess Medical Center, Boston, MA | 41 | 9.8 | 4.9 | 2.4 | 2.4 | 0.0 | 14.6 | 0.0 | 9.8 | -- | 2.4 | 7.3 | 9.8 |
| Children's Hospital, Washington, DC | 28 | 17.9 | 17.9 | 21.4 | 0.0 | 0.0 | 28.6 | 0.0 | 17.9 | -- | 3.6 | 7.1 | 28.6 |
| University of North Carolina Hospital, Chapel Hill, NC | 49 | 22.4 | 34.7 | 26.5 | 14.3 | 2.0 | 36.7 | 0.0 | 20.4 | -- | 16.3 | 16.3 | 46.9 |
| Dekalb Medical Center, Decatur, GA | 52 | 32.7 | 11.5 | 11.5 | 1.9 | 0.0 | 26.9 | 0.0 | 17.3 | -- | 7.7 | 3.8 | 28.8 |
| University of Louisville Hospital, Louisville, KY | 48 | 22.9 | 10.4 | 4.2 | 8.3 | 0.0 | 20.8 | 0.0 | 6.3 | -- | 2.1 | 6.3 | 18.8 |
| University of South Alabama, Mobile, AL | 58 | 17.2 | 24.1 | 17.2 | 6.9 | 0.0 | 37.9 | 0.0 | 13.8 | -- | 13.8 | 12.1 | 39.7 |
| Mount Sinai Medical Center, Miami Beach, FL | 27 | 18.5 | 33.3 | 25.9 | 22.2 | 0.0 | 29.6 | 0.0 | 29.6 | -- | 25.9 | 7.4 | 48.1 |

I, intermediate resistance; R, resistant; TMP/SMX, trimethoprim/sulfamethoxazole.

South and Southeast. The lowest overall rate of ceftriaxone resistance was 2.9%; the highest was 48.1%. With erythromycin, overall rates of resistance were 3.8% to 43.8%. With tetracycline, chloramphenicol, and TMP/SMX, the rates were 5.7% to 29.6%, 0.0% to 25.9%, and 11.9% to 63.2%, respectively. In general, the highest rates of resistance with most antimicrobial classes were observed in the same centers.

Among the 34 medical centers, 24 had participated in a similar study 3 years before (4). Because the sampling period, the nature of the patients, and the test methods were the same, resistance rates obtained with selected antimicrobial drugs were compared at the 24 centers for the two study periods (Table 6). In 19 of 24 centers, penicillin resistance rates (intermediate and resistant) increased by at least 2% during the 3-year interval. In six cases, the rate of increase was statistically significant (Table 6). The number of centers in which rates of resistance increased by >2% with other selected antimicrobial drugs over the 3-year period were ceftriaxone 13, erythromycin 21, tetracycline 20, chloramphenicol 12, and TMP/SMX 16. In most cases, resistance rates increased; however, with certain antimicrobial agents (i.e., ceftriaxone, TMP/SMX, and chloramphenicol), resistance rates remained the same or decreased. Three centers (Texas Children's Hospital, UNC Hospital, and the University of South Alabama Medical Center) had statistically significant increased rates of resistance to nearly every class of antimicrobial drug.

### Conclusions

A total of 1,601 clinical isolates of *S. pneumoniae* obtained from November 1997 to April 1998 from patients in 34 U.S. medical centers were characterized with respect to the in vitro activity of 23 antimicrobial drugs. The overall rate of penicillin resistance was 29.5% (17.4% with intermediate resistance; 12.1% fully resistant). Penicillin, amoxicillin, and amoxicillin/clavulanate had essentially equivalent MICs for the pneumococcal isolates examined. Among the cephalosporins tested, the rank order of activity based on a comparison of MICs was ceftriaxone > cefpodoxime = cefuroxime > cefprozil > cefixime > cefaclor = loracarbef > ceftibuten. NCCLS has developed MIC interpretive breakpoints for two of these compounds, ceftriaxone and cefuroxime. On the basis of these breakpoints, overall resistance rates were 14.9% with ceftriaxone (10.9% intermediate, 4.0% resistant) and 23.3% with cefuroxime (1.3% intermediate and 22.0% resistant). Although no NCCLS-approved breakpoints have been developed for cefaclor, loracarbef, and ceftibuten versus *S. pneumoniae*, the high MICs we obtained with these drugs indicate that they would be poor choices for treating pneumococcal infections.

We observed a direct relationship between penicillin activity and the activity of all the other ß-lactam drugs we examined. This relationship, which has been observed by others, results from the principal mechanism of penicillin resistance in *S. pneumoniae*, alterations in penicillin binding proteins (4-6). The same proteins to which penicillin binds are necessary for the expression of the activity of all other ß-lactam antimicrobial drugs (8-11).

Among the macrolides examined, clarithromycin was generally one dilution more active than erythromycin, which in turn was one dilution more active than azithromycin. However, since NCCLS MIC interpretive breakpoints differ for these agents and given the actual distribution of MICs, overall rates of resistance for these three agents were similar (19.0%).

Two previous studies have indicated that macrolide resistance with *S. pneumoniae* exists primarily in one of two forms: strains with altered ribosomal targets due to expression of the *ermAM* gene and strains with an active efflux pump due to expression of the *mefE* gene (13,14). *ErmAM*-positive isolates have constitutive expression of resistance and typically have high levels of resistance to both clindamycin (MICs $\geq$8 µg/ml) and erythromycin (MICs $\geq$64 µg/ml). Efflux mutants are susceptible to clindamycin (MICs $\leq$0.25 µg/ml) and typically have erythromycin MICs 1 to 32 µg/ml.

In a recent survey in Canada, approximately 60% of macrolide-resistant strains of *S. pneumoniae* appeared to be efflux mutants (19). In our study, 214 (70.8%) of 302 macrolide-resistant strains were characterized by the efflux phenotype. When a random sample of 35 of these isolates was examined by PCR, 31 (88.6%) lacked the *ermAM* gene but had the *mefE* gene responsible for efflux. Among the remaining four strains in this group that were characterized at a molecular level, one was *ermAM* positive and *mefE* negative, two were positive for both *ermAM* and *mefE,* and one was negative for all

Table 6. Resistance rate comparison of selected antimicrobial drugs for 24 medical centers, by study period

| Medical center | No. of isolates | Study period | Penicillin % I | Penicillin % R | Ceftriaxone % I | Ceftriaxone % R | Erythromycin % I | Erythromycin % R | Tetracycline % I | Tetracycline % R | Chloramphenicol % I | Chloramphenicol % R | TMP/SMX % I | TMP/SMX % R |
|---|---|---|---|---|---|---|---|---|---|---|---|---|---|---|
| Children's Hospital & Medical | 37 | 1994-95 | 21.6 | 13.5 | 8.1 | 8.1 | 0.0 | 5.4 | 0.0 | 18.9 | -- | 8.1 | 10.8 | 37.8 |
| Center, Seattle, WA | 50 | 1997-98 | 26.0 | 12.0 | 10.0 | 8.0 | 0.0 | 30.0[a] | 0.0 | 24.0 | -- | 10.0 | 8.0 | 34.0 |
| Denver Health, Denver, CO | 62 | 1994-95 | 11.3 | 3.2 | 1.6 | 0.0 | 0.0 | 3.2 | 0.0 | 3.2 | -- | 0.0 | 6.5 | 9.7 |
| | 26 | 1997-98 | 7.7 | 7.7 | 11.5 | 0.0 | 0.0 | 7.7 | 0.0 | 11.5 | -- | 11.5[a] | 3.8 | 15.4 |
| Good Samaritan Medical | 57 | 1994-95 | 21.1 | 19.3 | 21.1 | 7.0 | 0.0 | 12.3 | 0.0 | 14.0 | -- | 5.3 | 10.5 | 19.3 |
| Center, Phoenix, AZ | 54 | 1997-98 | 11.1 | 29.6 | 24.1 | 11.1 | 0.0 | 35.2[a] | 0.0 | 20.4 | -- | 5.6 | 11.1 | 38.9 |
| Texas Children's Hospital, | 63 | 1994-95 | 9.5 | 15.9 | 9.5 | 6.3 | 0.0 | 22.2 | 0.0 | 9.5 | -- | 7.9 | 7.9 | 30.2 |
| Houston, TX | 48 | 1997-98 | 39.6 | 25.0[a] | 25.0 | 10.4[a] | 0.0 | 43.8[a] | 2.1 | 22.9[a] | -- | 14.6 | 20.8 | 41.7 |
| Parkland Health & Hospital | 58 | 1994-95 | 8.6 | 13.8 | 8.6 | 5.2 | 0.0 | 6.9 | 0.0 | 5.2 | -- | 5.2 | 15.5 | 12.1 |
| System, Dallas, TX | 36 | 1997-98 | 19.4 | 11.1 | 11.1 | 2.8 | 0.0 | 27.8[a] | 0.0 | 22.2[a] | -- | 11.1 | 16.7 | 22.2 |
| Mayo Clinic, Rochester, MN | 35 | 1994-95 | 2.9 | 11.4 | 5.7 | 5.7 | 0.0 | 8.6 | 2.9 | 8.6 | -- | 5.7 | 8.6 | 22.9 |
| | 48 | 1997-98 | 16.7 | 6.3 | 6.3 | 4.2 | 0.0 | 20.8 | 2.1 | 10.4 | -- | 6.3 | 10.4 | 16.7 |
| Children's Hospital, | 65 | 1994-95 | 20.0 | 13.8 | 6.2 | 6.2 | 0.0 | 18.5 | 0.0 | 6.2 | -- | 7.7 | 6.2 | 29.2 |
| Milwaukee, WI | 55 | 1997-98 | 12.7 | 7.3 | 7.3 | 3.6 | 0.0 | 10.9 | 1.8 | 5.5 | -- | 3.6 | 12.7 | 14.5 |
| Evanston Hospital, | 49 | 1994-95 | 10.2 | 4.1 | 6.1 | 2.0 | 0.0 | 8.2 | 0.0 | 4.1 | -- | 4.1 | 4.1 | 16.3 |
| Evanston, IL | 35 | 1997-98 | 11.4 | 2.9 | 2.9 | 0.0 | 0.0 | 14.3 | 0.0 | 5.7 | -- | 0.0 | 5.7 | 8.6 |
| Rush-Presbyterian St. Luke's | 41 | 1994-95 | 19.5 | 14.6 | 4.9 | 14.6 | 0.0 | 17.1 | 0.0 | 7.3 | -- | 12.2 | 9.8 | 29.3 |
| Medical Center, Chicago, IL | 41 | 1997-98 | 14.6 | 4.9 | 4.9 | 2.4 | 0.0 | 19.5 | 0.0 | 17.1 | -- | 7.3 | 2.4 | 19.5 |
| Clarian Health Methodist | 63 | 1994-95 | 17.5 | 3.2 | 6.3 | 1.6 | 0.0 | 7.9 | 0.0 | 3.2 | -- | 1.6 | 14.3 | 14.3 |
| Hospital, Indianapolis, IN | 55 | 1997-98 | 18.2 | 7.3 | 3.6 | 0.0 | 1.8 | 16.4 | 0.0 | 7.3 | -- | 3.6 | 10.9 | 12.7 |
| Washington University, | 57 | 1994-95 | 19.6 | 5.4 | 8.8 | 3.5 | 0.0 | 8.9 | 0.0 | 7.1 | -- | 5.4 | 7.1 | 12.5 |
| St. Louis, MO | 55 | 1997-98 | 12.7 | 16.4 | 14.5 | 3.6 | 0.0 | 12.7 | 0.0 | 9.1 | -- | 5.5 | 18.2 | 12.7 |
| Henry Ford Health System, | 63 | 1994-95 | 17.5 | 1.6 | 1.6 | 4.8 | 0.0 | 6.3 | 0.0 | 4.8 | -- | 3.2 | 7.9 | 12.7 |
| Detroit, MI | 60 | 1997-98 | 21.7 | 8.3 | 10.0 | 3.3 | 0.0 | 10.0 | 0.0 | 8.3 | -- | 6.7 | 15.0 | 10.0 |
| The Cleveland Clinic Foun- | 42 | 1994-95 | 4.8 | 14.3 | 4.8 | 9.5 | 0.0 | 11.9 | 0.0 | 9.5 | -- | 7.1 | 11.9 | 26.2 |
| dation, Cleveland, OH | 60 | 1997-98 | 10.0 | 13.3 | 11.7 | 3.3 | 0.0 | 20.0 | 1.7 | 10.0 | -- | 6.7 | 11.7 | 15.0 |
| Temple University Hospital, | 47 | 1994-95 | 2.1 | 0.0 | 2.1 | 0.0 | 0.0 | 2.1 | 0.0 | 0.0 | -- | 0.0 | 4.3 | 4.3 |
| Philadelphia, PA | 42 | 1997-98 | 7.1 | 14.3[a] | 4.8 | 9.5[a] | 2.4 | 9.5 | 0.0 | 7.1 | -- | 4.8 | 2.4 | 9.5 |
| Geisinger Medical Center, | 57 | 1994-95 | 10.5 | 10.5 | 5.3 | 5.3 | 0.0 | 5.3 | 1.8 | 8.8 | -- | 7.0 | 5.3 | 10.5 |
| Danville, PA | 49 | 1997-98 | 30.6 | 8.2[a] | 12.2 | 0.0 | 0.0 | 20.4[a] | 0.0 | 20.4 | -- | 12.2 | 10.2 | 14.3 |
| SUNY Health Science Center, | 23 | 1994-95 | 8.7 | 0.0 | 0.0 | 0.0 | 0.0 | 8.7 | 0.0 | 4.3 | -- | 0.0 | 4.3 | 4.3 |
| Syracuse, NY | 50 | 1997-98 | 12.0 | 8.0 | 8.0 | 2.0 | 0.0 | 8.0 | 0.0 | 8.0 | -- | 6.0 | 12.0 | 8.0 |
| University of Rochester | 58 | 1994-95 | 5.2 | 5.2 | 5.2 | 1.7 | 0.0 | 6.9 | 0.0 | 10.3 | -- | 5.2 | 5.2 | 13.8 |
| Medical Ctr., Rochester, NY | 50 | 1997-98 | 10.0 | 10.0 | 10.0 | 2.0 | 2.0 | 10.0 | 0.0 | 8.0 | -- | 6.0 | 8.0 | 14.0 |
| Columbia Presbyterian Medical | 64 | 1994-95 | 6.3 | 6.3 | 4.7 | 4.7 | 0.0 | 4.7 | 0.0 | 3.1 | -- | 0.0 | 10.9 | 10.9 |
| Center, New York, NY | 53 | 1997-98 | 18.9 | 1.9 | 3.8 | 0.0 | 0.0 | 3.8 | 0.0 | 7.5 | -- | 5.7 | 5.7 | 7.5 |
| Hartford Hospital, | 61 | 1994-95 | 3.31 | 4.9 | 1.6 | 4.9 | 0.0 | 3.3 | 0.0 | 0.0 | -- | 0.0 | 0 | 8.2 |
| Hartford, CT | 51 | 1997-98 | 17.6 | 9.8[a] | 9.8 | 0.0 | 2.0 | 5.9 | 0.0 | 7.8[a] | -- | 2.0 | 11.8 | 17.6 |
| Children's Hospital, | 60 | 1994-95 | 16.7 | 6.7 | 5.0 | 3.3 | 0.0 | 13.3 | 0.0 | 3.3 | -- | 0.0 | 6.7 | 15.0 |
| Washington, DC | 28 | 1997-98 | 17.9 | 17.9 | 21.4 | 0.0 | 0.0 | 28.6 | 0.0 | 17.9[a] | -- | 3.6 | 7.1 | 28.6 |
| University of North Carolina | 60 | 1994-95 | 21.7 | 10.0 | 8.3 | 3.3 | 0.0 | 10.0 | 0.0 | 8.3 | -- | 6.7 | 16.7 | 25.0 |
| Hospital, Chapel Hill, NC | 49 | 1997-98 | 22.4 | 34.7[a] | 26.5 | 14.3[a] | 2.0 | 36.7[a] | 0.0 | 20.4 | -- | 16.3 | 16.3 | 46.9[a] |
| Dekalb Medical Center, | 61 | 1994-95 | 16.4 | 19.7 | 13.1 | 13.1 | 0.0 | 23.0 | 0.0 | 11.5 | -- | 9.8 | 1.6 | 27.9 |
| Decatur, GA | 52 | 1997-98 | 32.7 | 11.5 | 11.5 | 1.9 | 0.0 | 26.9 | 0.0 | 17.3 | -- | 7.7 | 3.8 | 28.8 |
| University of South | 68 | 1994-95 | 13.2 | 7.4 | 5.9 | 2.9 | 1.5 | 14.7 | 0.0 | 4.4 | -- | 0.0 | 2.9 | 25.0 |
| Alabama, Mobile, AL | 58 | 1997-98 | 17.2 | 24.1[a] | 17.2 | 6.9[a] | 0.0 | 37.9[a] | 0.0 | 13.8 | -- | 13.88 | 12.1 | 39.7[a] |
| Mount Sinai Medical Center, | 17 | 1994-95 | 29.4 | 23.5 | 5.9 | 17.6 | 0.0 | 5.9 | 0.0 | 17.6 | -- | 11.8 | 23.5 | 23.5 |
| Miami Beach, FL | 27 | 1997-98 | 18.5 | 33.3 | 25.9 | 22.2 | 0.0 | 29.6 | 0.0 | 29.6 | -- | 25.9 | 7.4 | 48.1 |

[a]Statistically significant change in resistance rates (I + R) from 1994-95 to 1997-98; p value:50.05.
I, intermediate; R, resistant; TMP/SMX, trimethoprim/sulfamethoxazole.

macrolide-resistance markers. Based on their phenotype (i.e., erythromycin MICs ≥64 µg/ml, with clindamycin MICs ≥8 µg/ml), 83 (27.5%) of the 302 macrolide-resistant strains appeared to have constitutive expression of *ermAM*-mediated methylation of ribosomal targets. All 38 randomly selected isolates from this group were *ermAM* positive; 12 were also *mefE* positive by PCR.

Five macrolide-resistant strains were not readily placed into either the efflux or the target modification groups based on phenotype. Two strains with high-level clindamycin resistance but erythromycin MICs 2 and 32 µg/ml were *ermAM* positive but *mefE* negative. Three strains with erythromycin MICs $\geq$64 had clindamycin MICs 0.5, 1, and 4 µg/ml. One of these strains, which had a clindamycin MIC of 4 and a high erythromycin MIC, appeared to be an efflux mutant since it was *mefE* positive but *ermAM* negative. The mechanism of resistance in the last two isolates in this group is unclear. Both isolates were negative by PCR for *ermAM* and *mefE*, as well as other genetic markers for macrolide and lincosamide resistance in gram-positive bacteria.

Several conclusions can be drawn from these observations. Macrolide resistance in *S. pneumoniae* is nearly always characterized either by efflux or ribosomal target modifications. The phenotype of isolates in both categories is highly predictable. Efflux mutants usually have erythromycin MICs 1 - 32 µg/ml and clindamycin MICs <0.25 µg/ml. Target modified strains are characterized by constitutive expression of resistance and typically have erythromycin MICs $\geq$64 µg/ml and clindamycin MICs $\geq$8 µg/ml. Although rare exceptions to these patterns exist, clindamycin activity, as assessed by an MIC determination, is a reliable indicator of the nature of macrolide resistance.

These results show that therapeutic efficacy can be predicted for patients with pneumococcal infections who are treated with macrolides. Using current NCCLS interpretive criteria, 18% to 19% of clinical isolates of *S. pneumoniae* in the United States would be categorized as resistant. Approximately three-fourths of the resistant strains, however, are efflux mutants and have mid-range resistant macrolide MICs. Patients infected with *mefE* versus *ermAM*-positive strains of *S. pneumoniae* may differ in their response to macrolide therapy. The answer to this important question would define true clinical rates of macrolide resistance. If *mefE* strains respond to therapy with macrolide drugs, then true macrolide resistance rates may not be 18% to 19%, but closer to 4% to 5%, and high-level clindamycin MICs could be used to accurately identify strains highly resistant to macrolide drugs.

Multidrug resistance was noted with 16.0% of 1,601 isolates, a substantial increase over the 9.1% prevalence of multidrug resistant *S. pneumoniae* reported in our 1994-95 study (4). Among multidrug resistant strains, four resistance patterns were the most common: penicillin and TMP/SMX (n = 111); penicillin, macrolide, and chloramphenicol (n = 75); penicillin, macrolide, tetracycline, and TMP/SMX (n = 57); and penicillin, macrolide, tetracycline, TMP/SMX, and chloramphenicol (n = 87). No obvious clustering of multidrug resistant strains was observed in either individual medical centers or geographic regions.

That we found resistance to ß-lactam antimicrobial agents and numerous non-ß-lactam agents in the same organism is of interest. The mechanisms of resistance to the different antimicrobial classes are distinct and do not appear to be linked genotypically. One explanation, therefore, for the increasing prevalence of multidrug resistant strains is clonal spread of organisms resistant to more than one class of antimicrobial drugs. Under such circumstances, regardless of the antimicrobial drug used to select for resistance, multidrug resistant organisms may become more prevalent. Clonal spread warrants further study using appropriate techniques for establishing clonal relationships among multiple geographically distinct isolates of *S. pneumoniae*.

### Acknowledgments

We thank Kay Meyer for excellent secretarial assistance. We also thank the following for providing clinical isolates of *Streptococcus pneumoniae:* Carla Clausen, Susan Rossmann, Paul Southern, Michael Wilson, Michael Saubolle, John Washington, Michael Dunne, Gerald Denys, Melodie Beard, Tom Thompson, Sue Kehl, Frank Cockerill, Pat Murray, Ann Robinson, Betz Forbes, Dwight Hardy, Phillis Della-Latta, Allan Truant, Joe Campos, Paul Bourbeau, Peter Gilligan, Bob Jerris, Kim Chapin, and Sue Sharp.

This study was funded by a grant from Abbott Laboratories, which produces clarithromycin.

Dr. Doern is Director of the Clinical Microbiology Laboratories at the University of Iowa College Hospital and Clinics and Professor in the Department of Pathology in the medical school. His research interests focus on antibiotic resistance among bacterial pathogens. He has been Chair of the Clinical Microbiology Division of the American Society for Microbiology and a member of the Antimicrobial Subcommittee of the National Committee for Clinical Laboratory Standards.

### Appendix

Isolates were frozen at -70°C at the University of Iowa College of Medicine until further characterization. After two subcultures, the identity of isolates was confirmed as *S. pneumoniae* by conventional tests and criteria. MICs were determined in Mueller-Hinton broth supplemented with 3% lysed horse blood, according to the broth microdilution method recommended by the National Committee for Clinical Laboratory Standards (NCCLS) (15). Microdilution trays (final volume 100 µl per well) were inoculated with approximately 5 x $10^5$ colony-forming units (CFU)/ml (final concentration) of test organism and incubated 22 to 24 hours at 35°C in ambient air before MICs were determined.

*S. pneumoniae* ATCC 49619 and *Haemophilus influenzae* ATCC 49247, ATCC 49766, and ATCC 10211 were used as controls. Calculations of the percentages of isolates resistant to individual agents were based on the most recent MIC interpretive criteria published by NCCLS (16).

Selected isolates were examined for mutations in the quinolone resistance-determining region of the *parC* and *par E* genes encoding the C and E subunits of topoisomerase IV, respectively, and the quinolone resistance-determining region of the *gyrA* gene encoding for the A subunit of DNA gyrase. Polymerase chain reaction (PCR) amplification with subsequent sequencing of PCR products was done by using the method and primers described by Pan et al. (17) and Perichon et al. (18). Other isolates were screened for various macrolide resistance determinants by PCR amplification as described by Shortridge et al. (13). Primers were chosen by using Oligo 5.0 software (NBI, Plymouth, MI) from sequences deposited in Genbank. Strains negative for both *mefE* (efflux mutants) and *ermAM* (ribosome methylase) were characterized further for the presence of other methylase genes (*ermA* and *ermC*), erythromycin esterase (*ereA* and *ereB*), MS efflux (*msrA*) and the gene for lincosamide resistance (*linA*), again as described by Shortridge et al. (13).

### References

1. Jorgensen JH, Doern GV, Maher LA, Howell AW, Redding JS. Antimicrobial resistance among respiratory isolates of *Haemophilus influenzae, Moraxella catarrhalis*, and *Streptococcus pneumoniae* in the United States. Antimicrob Agents Chemother 1990;34:2075-80.
2. Spika JS, Acklam RR, Plikaytis BD, Oxtoby MG, the Pneumococcal Surveillance Working Group. Antimicrobial resistance of *Streptococcus pneumoniae* in the United States, 1979-1987. J Infect Dis 1991;163:1273-8.
3. Barry AL, Pfaller MA, Fuchs PC, Packer RR. In vitro activities of 12 orally administered antimicrobial agents against four species of bacterial respiratory pathogens from U.S. medical centers in 1992 and 1993. Antimicrob Agents Chemother 1994;38:2419-25.
4. Doern GV, Brueggemann A, Holley HP Jr, Rauch AM. Antimicrobial resistance of *Streptococcus pneumoniae* recovered from outpatients in the United States during the winter months of 1994 to 1995: results of a 30-center national surveillance study. Antimicrob Agents Chemother 1996;40:1208-13.
5. Doern GV, Pfaller MA, Kugler K, Freeman J, Jones RN. Prevalence of antimicrobial resistance among respiratory tract isolates of *Streptococcus pneumoniae* in North America: 1997 results from the SENTRY Antimicrobial Surveillance Program. Clin Infect Dis 1998;27:764-70.
6. Jones RN, Pfaller MA, Doern GV. Comparative antimicrobial activity of trovafloxacin tested against 3,049 *Streptococcus pneumoniae* isolates from the 1997-1998 respiratory infection season. Diagn Microbiol Infect Dis 1998;32:119-26.
7. Thornsberry C, Brown SD, Yee C, Bouchillon SK, Marler JK, Rich T. Increasing penicillin resistance in *Streptococcus pneumoniae* in the U.S. Infections in Medicine Supplement 1993;93:15-24.
8. Grebe T, Hakenbeck R. Penicillin binding proteins 2b and 2x of *Streptococcus pneumoniae* are primary resistance determinants for different classes of ß-lactam antibiotics. Antimicrob Agents Chemother 1996;40:829-34.
9. Jamin M, Hakenbeck R, Frere JM. Penicillin binding protein 2x as a major contributor to intrinsic ß-lactam resistance of *Streptococcus pneumoniae*. FEBS 1993;331:101-4.
10. Laible G, Hekenback R. Five independent combinations of mutations can result in low-affinity penicillin-binding protein 2x of *Streptococcus pneumoniae*. J Bacteriol 1991;173:6986-90.
11. Markiewicz Z, Tomasz A. Variation in penicillin-binding protein patterns of penicillin-resistant clinical isolates of pneumococci. J Clin Microbiol 1989;27:405-10.
12. Kaplan SL, Mason EO Jr. Management of infections due to antibiotic-resistant *Streptococcus pneumoniae*. Clin Microbiol Rev 1998;11:628-44.
13. Shortridge VD, Flamm RK, Ramer N, Beyer J, Tanaka SK. Novel mechanism of macrolide resistance in *Streptococcus pneumoniae*. Diagn Microbiol Infect Dis 1996;26:73-8.
14. Sutcliffe J, Tait-Kamradt A, Wondrack L. *Streptococcus pneumoniae* and *Streptococcus pyogenes* resistant to macrolides but sensitive to clindamycin: a common resistance pattern mediated by an efflux system. Antimicrob Agents Chemother 1996;40:1817-24.
15. National Committee for Clinical Laboratory Standards. Methods for dilution antimicrobial susceptibility tests for bacteria that grow aerobically, 4th ed. Approved standard M7-A4. Wayne (PA): The Committee; 1997.
16. National Committee for Clinical Laboratory Standards. Performance standards for antimicrobial susceptibility testing. Eighth informational supplement, M100-S8. Wayne (PA): The Committee; 1997.
17. Pan X-S, Ambler J, Mehtar S, Fisher LM. Involvement of topoisomerase IV and NDA gyrase as ciprofloxacin targets in *Streptococcus pneumoniae*. Antimicrob Agents Chemother 1996;40:2321-6.
18. Perichon B, Tankovic J, Courvalin P. Characterization of a mutation in the *par E* gene that confers fluoroquinolone resistance in *Streptococcus pneumoniae*. Antimicrob Agents Chemother 1997;41:1166-7.
19. Johnston NJ, De Azavedo JC, Kellner JD, Low DE. Prevalence and characterization of the mechanisms of macrolide, lincosamide, and streptogramin resistance in isolates of *Streptococcus pneumoniae*. Antimicrob Agents Chemother 1998;42:2425-6.
